# Two-Field Excitation for Contactless Inductive Flow Tomography

**DOI:** 10.3390/s24144458

**Published:** 2024-07-10

**Authors:** Max Sieger, Katharina Gudat, Rahul Mitra, Stefanie Sonntag, Frank Stefani, Sven Eckert, Thomas Wondrak

**Affiliations:** Helmholtz-Zentrum Dresden-Rossendorf, Institute of Fluid Dynamics, Department Magnetohydrodynamics, Bautzner Landstraße 400, 01328 Dresden, Germany

**Keywords:** liquid metal flow, inductive measurements, contactless inductive flow tomography, inverse problem, magnetohydrodynamics

## Abstract

Contactless inductive flow tomography (CIFT) is a flow measurement technique allowing for visualization of the global flow in electrically conducting fluids. The method is based on the principle of induction by motion: very weak induced magnetic fields arise from the fluid motion under the influence of a primary excitation magnetic field and can be measured precisely outside of the fluid volume. The structure of the causative flow field can be reconstructed from the induced magnetic field values by solving the according linear inverse problem using appropriate regularization methods. The concurrent use of more than one excitation magnetic field is necessary to fully reconstruct three-dimensional liquid metal flows. In our laboratory demonstrator experiment, we impose two excitation magnetic fields perpendicular to each other to a mechanically driven flow of the liquid metal alloy GaInSn. In the first approach, the excitation fields are multiplexed. Here, the temporal resolution of the measurement needs to be kept as high as possible. Consecutive application by multiplexing enables determining the flow structure in the liquid with a temporal resolution down to 3 s with the existing equipment. In another approach, we concurrently apply two sinusoidal excitation fields with different frequencies. The signals are disentangled on the basis of the lock-in principle, enabling a successful reconstruction of the liquid metal flow.

## 1. Introduction

Contactless inductive flow tomography (CIFT) is able to reconstruct the global three-dimensional velocity field in electrically conducting fluids [1,2,3,4,5,6]. The method is based on the principle of induction by motion: very weak induced magnetic fields, **b**, originate from the fluid motion under the influence of a primary excitation magnetic field, **B_0_**, and can be precisely measured outside the fluid. As the structure of the induced magnetic field corresponds to the structure of the causative flow field, the solution of the associated linear inverse problem allows for reconstructing the velocity field. The concurrent use of more than one excitation magnetic field is necessary to fully reconstruct three-dimensional liquid metal flows. Due to its non-contact nature, CIFT enables reconstructing the time-dependent global vector field of the flow velocity, even in chemically aggressive and/or very hot liquid metals that are inaccessible by other techniques such as ultrasound-Doppler velocimetry or particle-image velocimetry. It can, therefore, be applied to a variety of tasks ranging from fundamental research [7] to industrial processes like continuous steel casting [8].

As the principle of induction is based on the cross product of velocity and the excitation magnetic field, **v** × **B_0_**, velocity components parallel to the magnetic field lines of the primary field cannot be probed. Certain geometries can still be investigated with only one axial primary field, e.g., axisymmetric poloidal flows [4], but other cases fail, e.g., a rotating cylinder in a homogeneous vertical excitation field [9]. Interestingly, it was demonstrated that only one primary magnetic field is sufficient to detect the relevant velocity field of liquid steel in molds of slab casters, because the dominating flow structure is almost two-dimensional [10]. On the contrary, flow structures in liquid metal Rayleigh–Bénard convection are three-dimensional [11,12,13]. For a reasonable reconstruction, the use of only one primary field direction necessitates additional assumptions, e.g., a non-rotating and poloidal dominated flow inside a cylindrical vessel [7]. While an unconditional reconstruction of three-dimensional flow structures requires two independently applied magnetic fields, the simultaneous use of more than one excitation magnetic field is not trivial, as the induced magnetic fields are scalar values that need to be decomposed with regard to the respective contribution of each excitation field.

A related inductive measurement technique is mutual inductance tomography (MIT), which is able to visualize the electrical conductivity distribution in a volume [14,15,16]. This method relies on direct AC induction in-between the different coils of an array, in which emitters and receivers are permanently interchanged. Electrically conductive or ferromagnetic objects close to the current emitter perturb its respective excitation magnetic field, and this perturbation is measured by the other induction coils of the array, which are currently in the receiving state. Contrary to CIFT, the ill-posed inverse problem is non-linear. This method can be used to detect, e.g., internal bleeding [17] or the distribution of gas and liquid metal in a pipe [18]. MIT systems can operate with one single or multiple frequencies [19,20,21,22]. In the latter case, sometimes also called “spectroscopic measurement” [23], measurements are performed by applying several frequencies one after another, i.e., not simultaneously.

As a proof-of-principle for the use of several excitation magnetic fields in CIFT, Stefani et al. applied two excitation magnetic fields with perpendicular directions to each other in an alternating manner to a mechanically driven liquid metal flow [6]. The dynamic range, i.e., ratio **b**/**B_0_**, was in the order of 10^−2^. Using this multiplexing scheme, the authors were successful in determining the flow structure in the fluid with a temporal resolution of 6 s. In the first part of this paper, we extend this approach to a higher dynamic range of more than three orders of magnitude, also aiming at a better temporal resolution of 3 s. Furthermore, we consider the concurrent use of two sinusoidal excitation fields with different frequencies, which lifts the time resolution limit of the multiplexing approach. The successful disentanglement of the signals on the basis of the lock-in principle, followed by the reconstruction of the 3D liquid metal flow structure, will be shown in the second part of the manuscript.

## 2. CIFT—Concept and Mathematical Basis

Based on the principle of induction by motion, CIFT enables us to determine the global flow field in electrically conducting fluids: the method takes advantage of the fact that the velocity field, **v**, of an electrically conductive fluid with a homogeneous electrical conductivity, σ, generates electric eddy currents in the same fluid under the influence of an external static magnetic field, **B_0_**. These eddy currents, in turn, induce their own flow-induced magnetic field, **b**, which is spatially and temporally structured according to its causing flow structure, **v**. Thus, **b** contains essential information from which the global flow field can be reconstructed. The key integral equation system for the induced magnetic field and the electric potential can be written as

(1) $$b\left(r\right)=\frac{{\sigma \mu }_{0}}{4\pi }{∭}_{V}\frac{\left(v\left(r^{\prime }\right)\times B_{0}\left(r^{\prime }\right)\right)\times \left(r-r^{\prime }\right)}{{\left|r-r^{\prime }\right|}^{3}}{\mathrm{dV}}^{\prime }-\frac{{\sigma \mu }_{0}}{4\pi }{∯}_{S}\varphi \left(r^{\prime }\right)\frac{n\left(r^{\prime }\right)\times \left(r-r^{\prime }\right)}{{\left|r-r^{\prime }\right|}^{3}}{\mathrm{dS}}^{\prime },$$

(2) $$\varphi \left(s\right)=\frac{1}{4\pi p\left(r\right)}{∭}_{V}\frac{\left(v\left(r^{\prime }\right)\times B_{0}\left(r^{\prime }\right)\right)\cdot \left(r-r^{\prime }\right)}{{\left|r-r^{\prime }\right|}^{3}}{\mathrm{dV}}^{\prime }-\frac{1}{4\pi p\left(r\right)}{∯}_{S}\varphi \left(r\prime \right)\frac{n\left(r^{\prime }\right)\cdot \left(r-r^{\prime }\right)}{{\left|r-r^{\prime }\right|}^{3}}{\mathrm{dS}}^{\prime },$$

where S=∂V is the boundary of the fluid volume V, μ_0_ is the magnetic permeability of vacuum, and **n**(r) is the normal vector at the location r on the surface. The term p depends on the shape of the boundary surface at position **r** [9]. The equations are valid for low magnetic Reynolds numbers,

(3) $$R_{m}=\mu_{0}\sigma l\nu <1,$$

wherein *l* and *v* are characteristic length scales and velocities. These integral equations are solved numerically by discretizing V and S with linear shape functions. In the inverse problem, the discretized velocity field $\tilde{v}$ can be reconstructed by minimizing the following quadratic functional:

(4) $$\underset{\tilde{v}}{\min}\left(\sum_{i=1}^{n}\|M_{i}\cdot \tilde{v}-{\tilde{b}}_{i}{\|}_{2}^{2}+\lambda \|A\tilde{v}{\|}_{2}^{2}+\lambda_{D}\|\nabla \cdot \tilde{v}{\|}_{2}^{2}\right),$$

where $M_{i}$ is the linear operator derived from the integral equation system in Equations (1) and (2) for the *i*-th applied magnetic field and ${\tilde{b}}_{i}$ is the corresponding vector containing the measurement of the magnetic field at the sensors. The parameter λ_D_ is chosen large to ensure the divergence-free condition of the reconstructed velocity and λ represents the Tikhonov regularization parameter with the according matrix A being used to mitigate the intrinsic non-uniqueness of the inverse problem. The optimal regularization parameter λ_opt_ is obtained by the L-curve method [6,24,25].

It is interesting to note that the integral equation system and inverse problem of CIFT are similar to magnetoencephalography [26,27], which is able to reconstruct active regions in the brain by measuring the magnetic field outside of the head, and magnetocardiography [28,29], which is able to reconstruct the current distribution in the heart muscle by measuring the magnetic field outside of the thorax. The sources of the magnetic field are the active neurons in the brain and active muscles in the heart, respectively. While, in both methods, the current distribution in the tissue has to be reconstructed from only of one magnetic field measurement, in the case of CIFT, excitation magnetic fields in different directions can be applied consecutively to obtain several magnetic field measurements for the same flow field for a better reconstruction of the flow.

## 3. Experimental Set-Up

### 3.1. General

**Figure 1:**
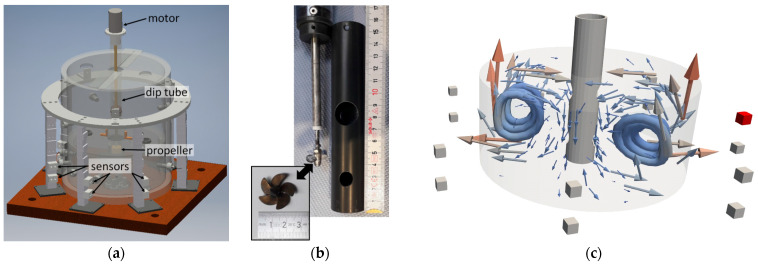
Experimental set-up: (**a**) technical drawing with main components marked, (**b**) close-up of the dip tube with inlets and outlets and propeller (inset), and (**c**) visualization of the stationary flow structure determined by the inlets and outlets in a blended vector and streamline representation including sensor positions (grey boxes, one sensor that is evaluated in detail in following is marked in red).

Experiments were carried out in a cylindrical vessel with a diameter of 230 mm, filled with the liquid metal alloy GaInSn up to a level of 100 mm (for a detailed description of the material properties of GaInSn, refer to [30]). The liquid metal was pumped by a propeller driven by a motor at the top of a dip tube (Figure 1a). A detailed view of the propeller position with respect to the inlet and outlet positions at the lower part of the dip tube is given in Figure 1b. Each experimental run started and ended with time intervals without stirring of 60 s each, which were necessary to determine the magnetic baseline for CIFT. In the actual flow phase of 60 s in the middle, the propeller drove the flow. The flow structure was stationary because the propeller was intaking and ejecting the liquid metal through defined inlets and outlets of the dip tube (Figure 1c). Therefore, a change in propeller speed only influenced the flow rate. Magnetic field sensors to detect the induced magnetic fields were mounted in fourteen locations around this cylinder. The sensors were located in seven angular positions equally distributed over the azimuth with a radial distance of about 180 mm to the center of the cylinder and at two heights of 14 and 50 mm above the bottom of the cylinder, as schematically shown in Figure 1c.

### 3.2. Excitation Magnetic Fields

**Figure 2:**
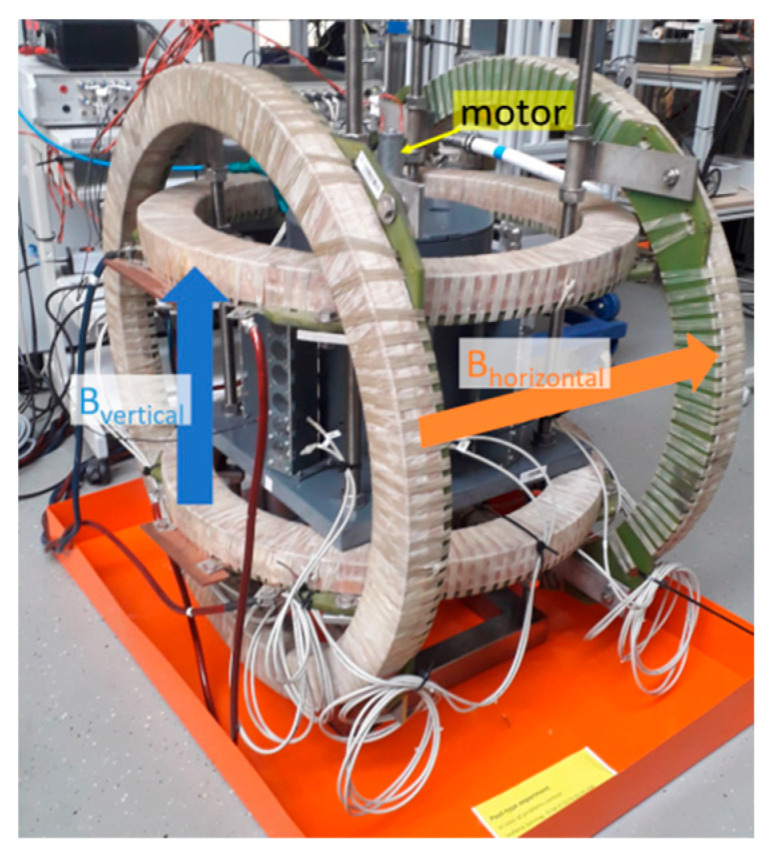
Photograph showing the liquid metal tank inside the two Helmholtz coil pairs for vertical and horizontal excitation magnetic fields.

Figure 2 depicts the Helmholtz-like coil pairs to create two excitation magnetic fields in horizontal and vertical direction, respectively. The horizontal coil pair had an inner radius of 305 mm and mutual spacing of 495 mm. The vertical coil pair had an inner radius of 265 mm and a mutual spacing of 370 mm. Both coil pairs were driven by separate *KEPCO* BOP20-50 GL current amplifiers. The excitation electric currents were chosen to create similar excitation magnetic fields in the order of 1 mT with respect to the saturation of the used magnetic field sensors. Utilizing a *Keysight* 33500B series 2-channel function generator, it was possible to apply different schemes for the excitation magnetic fields, that will be explained in detail in the following.

## 4. Consecutive Excitation

**Figure 3:**
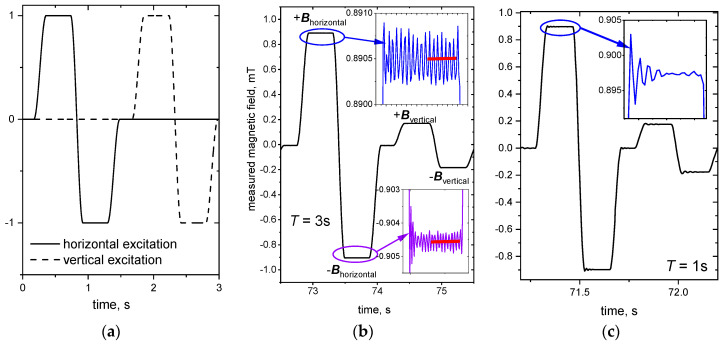
(**a**) General scheme of the consecutive CIFT two-field excitation (trapezoidal-shaped single profiles, total cycle length for both excitation fields 3 s, normalized amplitude). (**b**) Raw measurement data of induced magnetic field **b** for a total cycle length of 3 s for one exemplary sensor, marked red in Figure 1c. (**c**) Raw measurement data of induced magnetic field **b** for a total cycle length of 1 s for the same sensor. Insets show initial current source oscillations, red lines mark the area of data extraction in the second half of each plateau.

Following the approach of Stefani et al. [6], both excitation magnetic field directions were consecutively switched on and off according to the sequence shown in Figure 3a, i.e., either **B**_horizontal_ or **B**_vertical_ was on, but not both at the same time. This trapezoidal-shaped excitation scheme consisted of a positive and a negative plateau and was programmed with the *Keysight Benchlink Waveform Builder Pro 2019* to avoid sharp kinks. Commercial 1D fluxgate sensors (*Institut Dr. Foerster GmbH*, Reutlingen, Germany) were used to detect the induced magnetic fields, as they are suitable for measurements of magnetic fields in the range from 0.14 nT to 1.2 mT, and, in particular, they possess a high dynamic range **b**/**B_0_**. With respect to CIFT, fluxgate probes were previously used to measure induced magnetic fields caused by a static excitation magnetic field in Rayleigh–Bénard convection experiments [7], as well as in model experiments for continuous casting [10].

The beginning of each plateau is characterized by a transient phenomenon, see insets of Figure 3b,c, which become stronger with a decreasing cycle time (note that the changes in induced magnetic field in the inset of Figure 3c are 10 times larger than compared to Figure 3b). A period of 10 s was initially chosen for the excitation scheme, but this was gradually reduced to 5 s, 3 s, 2 s, and 1 s in the interest of improving the temporal resolution of the measurements. For a cycle time of less than 3 s, the oscillation effects superpose the actual measurement data already in the full duration of the plateau, so that the induced magnetic fields cannot be extracted any more. Therefore, with our equipment, the best possible temporal resolution with this excitation scheme is approximately 3 s per cycle, which corresponds to a single plateau duration of only 0.37 s, of which 0.07 s are attributed to the initial oscillations. The experiment with a cycle time of 3 s was chosen for further evaluation and flow reconstruction: An automatic algorithm was established to exclude the initial transient region and take only the average value of the second half of each plateau (red lines in Figure 3b inset) to extract the very small induced magnetic field values from the excitation fields’ background. The excitation field offsets in both plateaus cancel each other out during the averaging of each plateau pair according to,

(5) $$B_{o}^{+}+b^{+}-\left(B_{o}^{-}-b^{-}\right)=b^{+}+b^{-}=2\cdot b,$$

revealing one *demodulated* induced magnetic field value per period for the horizontal excitation field and one value per period for the vertical excitation field.

**Figure 4:**
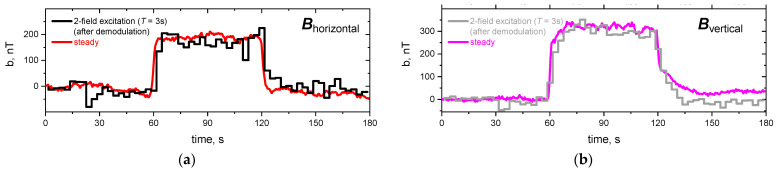
Induced magnetic fields for the sensor marked in red in Figure 1c, for (**a**) horizontally applied magnetic field from single steady (red) and consecutive two-field (black) excitation scheme, and for (**b**) vertically applied magnetic field from single steady (purple) and consecutive two-field (grey) excitation scheme. The demodulated values of the two-field excitation refer to the experiment with the lowest possible cycle time of 3 s.

Reference measurements were carried out with single steady (DC) excitation magnetic fields in the horizontal and vertical directions. All magnetic field measurements were corrected for the constant magnetic field contributions of Earth’s magnetic field and ferromagnetic parts in close vicinity to the experiment that are reflected as an offset in the raw data in the no-flow phase. Figure 4 depicts the induced magnetic field values at one particular sensor, marked in red in Figure 1c. This sensor was selected because it measures the strongest flow-induced magnetic field.

**Figure 5:**
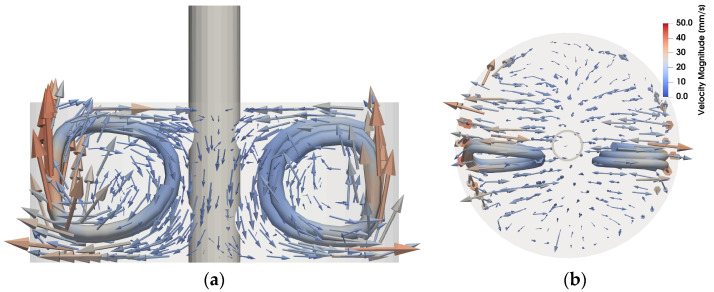
Reconstructed time-averaged velocity field of the consecutive excitation approach with a total cycle length of 3 s in a blended vector and streamline visualization: (**a**) side-view and (**b**) top-view.

The three phases of each experiment can directly be identified from Figure 4, which depicts the induced magnetic field for one exemplary sensor, marked red in Figure 1c, for the full time of the experiment: in the first and last 60 s the liquid metal was at rest and, therefore, no induced magnetic field contribution was measured. Within 60 s < *t* < 120 s the flow-induced magnetic field caused by the propeller driven flow was detected. As the flow is largely stationary, the measured induced magnetic field remained almost constant within the phase of flow. The difference in amplitude between the horizontal and vertical excitation fields was related to the very different projections of **v** onto the excitation magnetic field **B_0_**. The demodulated values of the two-field excitation followed the same general behavior and were in good agreement with the values of the respective steady excitation fields. The measured induced magnetic field in the order of a few hundred nT resulting from an excitation magnetic field in the order of 1 mT yields a dynamic measurement range of more than three orders of magnitude, which is a strong improvement compared to the previous results of Stefani et al. However, some stronger fluctuations could be identified in the signal, e.g., for **B**_vertical_ at around 32 s, being an indication that the automatic algorithm for extracting the demodulated values could be further improved. The demodulated induced magnetic field values were used to reconstruct the flow field in the liquid metal. Figure 5 gives the time average of the flow reconstruction for 60 s < *t* < 120 s in a blended vectorial and streamlined representation to highlight the stationary swirls on each side of the dip tube, induced by the inlet and outlet holes as expected (cf. Figure 1c). The mean magnitude of the flow velocity was around 12 mm/s, with a maximum of 40 mm/s for both upstreaming flows.

## 5. Concurrent Excitation

To overcome the restriction in the temporal resolution of the consecutive excitation (3 s in this study), simultaneous sinusoidal excitation schemes with varying frequencies were tested as a proof-of-concept. The previously used fluxgate sensors were replaced with in-house developed induction coils that have no intrinsic upper limit of detection, i.e., no saturation as for the fluxgate probes. Two types of induction coils are available, both are made of a 25 μm baked enamel wire on a polytetrafluoroethylene (PTFE) frame: absolute coils consist of a single winding with 340,000 turns and pick up the absolute value of the time-derivative of the spatial magnetic field with a sensitivity of 510 V/(T∙Hz), whereas gradiometric induction coils are composed of two separate windings of 2 × 160,000 turns, wound in opposite directions to detect the time derivative of the spatial magnetic field gradient in the axial direction of the induction coil with a sensitivity of 240 V/(T∙Hz). A detailed review on these sensors can be found in [31]. For a proof-of-concept, both types of sensors were used here simultaneously: the upper ring of sensors was equipped with absolute induction coils, while the lower ring contained gradiometric sensors, cf. Figure 1c. This approach is justified, because the measured radial induced magnetic field component was nearly the same at both heights, as previously determined for the fluxgate probes. The induction coils were connected to a precise 24-bit *Tasler* *LTT24* A/D converter (*Labortechnik Tasler GmbH*, Würzburg, Germany), capable of resolving a dynamic measurement range of up to six orders of magnitude, to record the induced magnetic fields with a high data acquisition rate.

The theoretical limit for the excitation frequency is given by the skin effect: an alternating magnetic field exponentially decays within an electrically conducting volume, being confined to a thin surface layer—the “skin” of the conductor—for high frequencies. The thickness δ of this surface layer, i.e., the penetration or skin depth, is mainly determined by the electrical conductivity, σ, of the material and the frequency, *f*, according to

(6) $$\delta =\sqrt{1/\left(\sigma \pi f\mu_{0}\right)}.$$

It should be noted that the skin depth does not indicate a distance at which the magnetic field suddenly drops from a constant plateau to zero, but specifies the exponential damping to 1/e ≈ 0.37. Taking this into account, the highest frequency that can be applied to penetrate the whole GaInSn volume can be calculated to be around 10 Hz, which corresponds to a skin depth of approximately 90 mm, i.e., almost the vessel’s radius. However, the subtle use of higher frequencies can be utilized to separate flow information in the center of the probed volume from regions close to the edge.

In the following, the case of *f*_horizontal_ = 1 Hz and *f*_vertical_ = 2 Hz is visualized and further evaluated. The contributions of the different frequencies of the concurrent excitation were demodulated on the basis of the lock-in principle, i.e., filtering the flow-induced magnetic field for the respective carrier frequencies that are known. The temporal resolution is determined by the lowest frequency; in our case, it is 1 s. In principle, this value could be improved easily by increasing both excitation frequencies, however, 1 s is sufficient for the envisaged application in Rayleigh–Bénard convection and other experiments.

**Figure 6:**
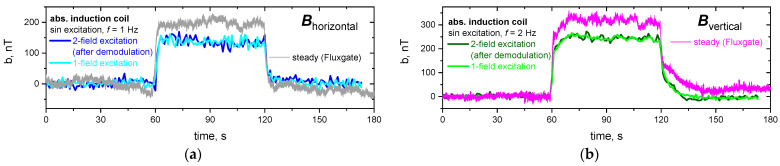
Induced magnetic field for the direct induction coil marked in red in Figure 1c, for a concurrent excitation with sinusoidal schemes with (**a**) *f*_horizontal_ = 1 Hz and (**b**) *f*_vertical_ = 2 Hz in comparison with the respective single-field excitation (AC) and in relation to the steady (DC) excitation measured with fluxgate probes (cf. Figure 4).

The demodulated contributions of the induced magnetic field for the simultaneous excitation approach with *f*_horizontal_ = 1 Hz and *f*_vertical_ = 2 Hz are compared with the respective values of the induced magnetic field for the individual excitation of each excitation direction in Figure 6: as before, the three intervals are directly recognizable for each experimental run with an almost constant plateau value of 60 s < *t* < 120 s due to the stationary flow conditions.

The demodulated sinusoidal signals show only minor disturbances and are in very good agreement with the respective individual one-field excitation experiments. The absolute values of the induced magnetic field for the concurrent approach are slightly lower than those for the consecutive excitation approach. The reason for this deviation is a slightly larger distance of about 6 mm of the induction coils to the liquid metal, which is taken into consideration during reconstruction. Therefore, the reconstructed flow field gives the same structure with stationary swirls one each side of the dip tube and is not depicted again. A multitude of frequency combinations in the range of 1–10 Hz, including, e.g., the “inverted” case *f*_horizontal_ = 2 Hz and *f*_vertical_ = 1 Hz, were successfully demodulated and give the same results as discussed for the presented frequency combination.

## 6. Conclusions

We present a proof-of-concept for the use of two excitation magnetic fields for contactless inductive flow tomography. The use of more than one excitation magnetic field is necessary to fully reconstruct three-dimensional liquid metal flows, but is not trivial, as the scalar values of the induced magnetic field at the sensors must be disentangled for each contribution of the excitation fields.

In our laboratory demonstration experiment, we applied two perpendicular excitation magnetic fields to a mechanically driven flow in the liquid metal alloy GaInSn. Two approaches for excitation were followed: firstly, the use of a consecutive application by multiplexing allowed for automatically disentangling the contributions of the induced magnetic fields for each excitation field up to a dynamic range of more than three orders of magnitude, enabling the reconstruction of the three-dimensional velocity vector field inside the vessel with a temporal resolution down to 3 s with the existing equipment. Secondly, simultaneously applying two sinusoidal excitation fields with different frequencies allowed for successfully disentangling the overlapping contributions on the basis of the lock-in principle. The liquid metal flow field can be reconstructed likewise. The two-field excitation approach enhances the applicability of CIFT for three-dimensional flows, e.g., liquid metal Rayleigh–Bénard convection experiments in the future.

## Data Availability

Dataset available on request from the authors.
